# The Role of Acute Intermittent Hypoxia in Neutrophil-Generated Superoxide, Sympathovagal Balance, and Vascular Function in Healthy Subjects

**DOI:** 10.3389/fphys.2017.00004

**Published:** 2017-01-23

**Authors:** Germana P. L. Almeida, Ivani C. Trombetta, Felipe X. Cepeda, Elaine Hatanaka, Rui Curi, Cristiano Mostarda, Maria C. Irigoyen, José A. S. Barreto-Filho, Eduardo M. Krieger, Fernanda M. Consolim-Colombo

**Affiliations:** ^1^Heart Institute do Hospital das Clínicas da Faculdade de Medicina da Universidade de São PauloSão Paulo, Brazil; ^2^Departamento de Medicina Clínica, Federal University of CearáCeará, Brazil; ^3^Graduate Program in Medicine, Uninove, Universidade Nove de JulhoSão Paulo, Brazil; ^4^Instituto de Ciências da Atividade Física e Esportes, Universidade Cruzeiro do SulSão Paulo, Brazil; ^5^Department of Physiology and Biophysics, Institute of Biomedical Sciences, University of São PauloSão Paulo, Brazil; ^6^Programa de Pós-Graduação em Saúde do Adulto e da Criança, Federal University of MaranhãoMaranhão, Brazil; ^7^Division of Cardiology, Federal University of SergipeSergipe, Brazil

**Keywords:** oxidative stress, sympathovagal balance, chemoreflex, O_2_ saturation, peripheral vascular resistance, blood pressure

## Abstract

**Introduction:** Recurrent hypoxia (HPX), a hallmark of the obstructive sleep apnea (OSA), impairs autonomic balance, and increases arterial blood pressure (BP). Oxidative stress is one of the mechanisms involved in these alterations. The cumulative effect of acute intermittent HPX and the chronicity may determine whether the response crosses the threshold from having protective value to pathology. However, the impact of acute intermittent HPX–reoxygenation on markers of oxidative stress in healthy individuals remains to be fully understood.

**Objective:** To analyze the effects of the acute intermittent HPX on the generation of neutrophil-derived superoxide, sympathovagal balance, and vascular function in healthy subjects.

**Methods:** We applied six cycles of intermittent HPX (10% O_2_ and 90% N_2_) for 5 min followed by 2 min of room-air in 15 healthy volunteers (34 ± 2 years; 22.3 ± 0.46 kg/m^2^), without OSA (polysomnography), during wakefulness. During the experimental protocol, we recorded O_2_ saturation, end-tidal CO_2_, heart rate (HR), systolic, and diastolic BP, cardiac output (CO) and peripheral resistance (PR). Cardiac sympathovagal balance was determined by HR variability analysis (low frequency and high frequency bands, LF/HF). Superoxide generation in polymorphonuclear neutrophil cells were established using relative luminescence units (PMNs RLU) at baseline (pre-HPX) and immediately after hypoxia induction (post-HPX6).

**Results:** The studied subjects had normal levels of BP, plasma glucose, lipid profile, and inflammatory marker (C-reactive protein). Acute intermittent HPX increased HR, systolic BP, CO, and decreased PR. Additionally, acute intermittent HPX increased PMNs RLU, measured post-HPX6 (470 ± 50 vs. 741 ± 135, *P* < 0.05). We found a similar increase in LF/HF post-HPX6 (0.91 ± 0.11 vs. 2.85 ± 0.40, *P* < 0.05). PR was diminished from pre-HPX to post-HPX6 (1.0 ± 0.03 vs. 0.85 ± 0.06, *P* < 0.05). Further analysis showed significant association between O_2_ saturation and PMNs RLU (*R* = −0.62, *P* = 0.02), and with LF/HF (*R* = −0.79, *P* = 0.02) post-HPX6. In addition, an association was found between PMNs RLU and PR post-HPX6 (*R* = 0.58, *P* = 0.04).

**Conclusion:** Acute exposure to intermittent HPX not only increased superoxide generation in neutrophils, but also impaired cardiac sympathovagal balance in healthy subjects. These data reinforce the role of intermittent HPX in superoxide generation on neutrophils, which may lead to an impairment in peripheral vascular resistance.

## Introduction

Intermittent hypoxia (HPX), defined as repeated episodes of hypoxia interspersed with episodes of normoxia, has been observed in different types of sleep-disordered breathing, including obstructive sleep apnea syndrome (OSA) (Lévy et al., 2008).

Repeated episodes of hypoxia elicit changes in a range of physiological responses (Lévy et al., 2008; Foster et al., 2010; Gilmartin et al., 2010; Tamisier et al., 2011). The cumulative effect of intermittent HPX and the chronicity of intermittent HPX may determine whether the response crosses the threshold from having protective value to pathology. Furthermore, the presence of clinical diseases and environmental conditions may interfere with the response to this challenge.

It is not known if acute intermittent HPX–reoxygenation are able to increase polymorphonuclear and peripheral blood mononuclear cell mobilization, enhance superoxide generation in neutrophils, and impair sympato-vagal control and vascular function in healthy volunteers. Indeed, the threshold from physiological to pathophysiological condition may be triggered by inflammation and oxidative stress. It has already been found that the continuum release of a number of pro-inflammatory cytokines by macrophages sensitizes neutrophils and macrophages, thus producing superoxide (Laskin et al., 2011). On the other hand, while the generation of active oxygen species by neutrophils is one of the defense mechanisms against foreign pathogens, in chronic condition it may lead to inflammation and diseases.

In a chronic state, autonomic and hemodynamic changes stimulate the intravascular production of superoxide (Schultz, 2009), promoting inflammation, and damage to the vascular integrity and endothelial cell function (Laskin et al., 2011). Moreover, among subjects with untreated OSA, the release of superoxide from circulating neutrophils, was markedly enhanced when compared to control subjects (Schulz et al., 2000). However, the effect of acute intermittent HPX on immune cells mobilization and activation in healthy humans has not been fully investigated.

OSA has been consistently associated with increased cardiovascular morbidity/mortality (Fu et al., 2016). There is growing evidence that OSA may be directly involved in multiple pathways associated with cardiovascular risk. In this context, autonomic dysfunction seems to play a major role (Trombetta et al., 2013). Recent studies have suggested that chronic and acute intermittent HPX increases sympathetic activation via peripheral chemoreceptors stimulation (Trombetta et al., 2013), acutely increasing arterial blood pressure (BP) (Foster et al., 2010; Gilmartin et al., 2010; Tamisier et al., 2011). Indeed, in OSA patients, regardless of other cardiovascular risk factors, the cumulative effect of the repetitive hypoxia/hypercapnia episodes has been found to lead to an autonomic imbalance shift toward sympathetic tone (Trombetta et al., 2010, 2013; Cepeda et al., 2015), proinflammatory response, (Yokoe et al., 2003; Ryan et al., 2005) endothelial dysfunction (Kato et al., 2000), and chronic oxidative stress (Schulz et al., 2000).

To avoid the effect of pathological chronicity interference and isolate the acute effect of intermittent HPX–reoxygenation in neutrophil-generated superoxide and sympathovagal balance, we chose to investigate the acute intermittent HPX–reoxygenation in in healthy subjects, which was to identify the implication of intermittent HPX as mediator in the genesis of oxidative stress, even in the absence of other pathophysiological changes.

In the present study, we tested the hypothesis that brief cycles of intermittent HPX–reoxygenation are able to increase polymorphonuclear and peripheral blood mononuclear cell mobilization, enhance superoxide generation in neutrophils, and impair sympato-vagal control and vascular function in healthy volunteers.

## Methods

### Study population

Fifteen healthy subjects from the community, with no clinical and laboratory evidence of disease, participated in the study. Women were studied during the proliferative phase of the reproductive cycle. Smokers and subjects presenting OSA (defined by an apnea-hypopnea index >5 events/hour of sleep by the polysomnography, Drager et al., 2005) were excluded. All subjects provided informed written consent, and the study was approved by the Ethics Committee of the Heart Institute of the University of São Paulo.

### Overnight polysomnography

In order to exclude OSA, all subjects underwent an overnight polysomnography, performed as previously described (Trombetta et al., 2013). Briefly, the polysomnography was undertaken using the EMBLA digital system (17 channels, EMBLA, Flaga hf. Medical Devices, Reykjavik, Iceland). The apnea-hypopnea index (AHI) was calculated as the total number of respiratory events (apneas plus hypopneas) per hour of sleep, while the presence of OSA was defined by an AHI ≥5 events/h.

### Laboratory measurements

For all volunteers, blood samples were collected from venous blood after 12 h of overnight fasting, according to standard laboratory techniques in the clinical laboratory of the Heart Institute, to determine total cholesterol, low-density lipoprotein cholesterol (LDL-c), high-density lipoprotein cholesterol (HDL-c), triglycerides, glucose, and C-reactive protein.

### Experimental protocol

#### Acute intermittent HPX

**Figure 1:**
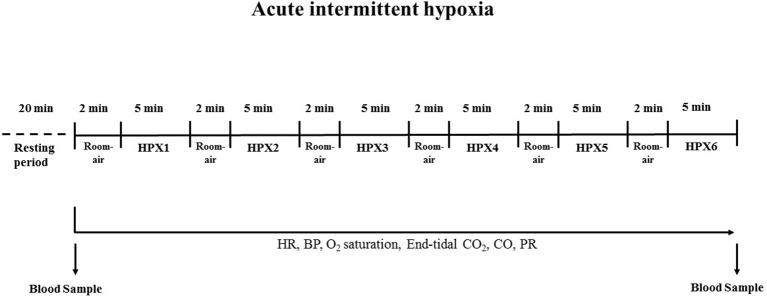
**Experimental protocol for evaluation of the effects of acute intermittent hypoxia (HPX, 10% O_**2**_ and 90% N_**2**_)**. HR, heart rate; BP, blood pressure; CO, cardiac output; PR, peripheral resistance.

All experiments were realized approximately at 8:00 a.m. Acute intermittent HPX protocol was performed while the subjects were awake after at least a 20 min of rest period in supine position in a quiet and temperature-controlled room. For the experimental protocol, the subjects completed a single day session of six cycles of breathing a gas mixture (10% O_2_, 90% N_2_) via a mouthpiece, for 5-min periods with intervening 2-min periods of room-air inspiration (Figure 1). A nose clip was used to ensure exclusive mouth breathing. The end-tidal carbon dioxide (CO_2_) and oxygen saturation were continuously monitored by a capnograph and pulse oximeter (Novametrix, model 7100 CO_2_ SMO ETCO_2_/SpO_2_ Monitor, Novametrix Medical Systems Inc.). The CO_2_ was titrated to maintain isocapnia. Non-invasive beat-to-beat arterial BP curves were continuously recorded by a finger photoplethysmography device (Finometer, Finometer Medical Systems BV, Arnhem, the Netherlands). Heart rate (HR) was evaluated by electrocardiography and arterial BP, cardiac output (CO) and peripheral vascular resistance (PR) were calculated using a BeatScope software and were analyzed as previously described (Barreto-Filho et al., 2003). Blood samples were collected before intermittent HPX (pre-HPX) and immediately after the completion of the protocol (post HPX 6) to perform white cell counting and analyze superoxide production at interval-period (approximately 45 min-period interval).

##### Polymorphonuclear neutrophils (PMNs) and peripheral blood mononuclear cell (PBMCs)

Polymorphonuclear (PMNs) and peripheral mononuclear cells (PBMCs) were isolated from the blood, as previously described (Jun et al., 2008), using a commercial gradient of Ficoll–Hypaque (Sigma-Aldrich Histopaque) and counted on a Neubauer chamber.

##### Superoxide generation in neutrophils determined by relative luminescence units (PMNs RLU)

Neutrophils were isolated from the blood (Böyum, 1968), using a commercial gradient of Ficoll–Hypaque (Histopaque). Lucigenin (1 mM) was added to neutrophil (2.5 × 10^6^ cells/ml) incubation medium when required. This method has been widely used to measure kinetics productions of superoxide by neutrophils (Hatanaka et al., 2006). Lucigenin releases energy in the form of light after being excited with superoxide anion. The assays were performed in a PBS buffer supplemented with CaCl_2_ (1 mM), MgCl_2_ (1.5 mM), and glucose (10 mM), at 37°C, in a final volume of 0.3 ml. Chemiluminescence response was monitored for 20 min, at 37°C, in a microplate luminometer (EG&G Berthold LB96V), before and after the addition of 16 ng of phorbol 12-myristate 13-acetate (PMA). Superoxide generation in polymorphonuclear neutrophil cells in relative luminescence units (PMNs RLU) was expressed as nanomoles of O_2_ per 2.5 × 10^6^ PMNs.

### Heart rate variability analysis

HR variability was analyzed in the frequency domains during the protocol, as previously described (Mello et al., 2012). The spectral bands for humans (very low frequency—VLF, 0.0–0.04 Hz; low frequency—LF, 0.04–0.15 Hz; and high frequency—HF, 0.15–0.4 Hz) were defined according to the literature (Pagani et al., 1986). The spectral power for the low- and high-frequency bands was calculated by power spectrum density integration within each frequency band width. Power density of each spectral component was calculated in normalized units. The powers in LF and HF for pulse interval were normalized by calculating the variance minus the power in VLF and were expressed in normalized units (nu). Sympathovagal balance was defined by the LF/HF ratio, expressed in nu. LF components of the R-R pulse interval variability was found to be markers of efferent sympathetic cardiac, whereas the HF component of the R-R pulse interval variability would reflect vagal modulation of the sinoatrial node (Pagani et al., 1986).

### Statistical analysis

The data are presented as the means ± standard error. The normal distribution of each variable was evaluated using the Kolmogorov-Smirnov test. The comparison of the cell counts in both groups was done by Wilcoxon's nonparametric test. We used one-way ANOVA for repeated measures followed by Tukey-Kramer. Test for multiple comparisons were used in hemodynamic, autonomic and blood samples data. Values of *P* < 0.05 were considered statistically significant.

## Results

Baseline characteristics are shown in Table 1. Fifteen healthy subjects (predominantly women) participated in the present study. The volunteers had normal BMI, BP, glucose, lipid profiles and CRP. In addition, all participants were within the normal range of the AHI, based on the polysomnography records (Table 1).

**Table 1:** **Characteristics of the studied population**.

	**All (*n* = 15)**
Age (y)	34 ± 2
Gender (male/female)	(6/9)
BMI (kg/m^2^)	22.30 ± 0.46
SBP (mmHg)	125 ± 4
DBP (mmHg)	79 ± 3
Heart rate (bpm)	70 ± 2
Total cholesterol (mg/dL)	160 ± 6
LDL-c (mg/dL)	98 ± 5
HDL-c (mg/dL)	46 ± 2
Triglycerides (mg/dL)	85 ± 9
Glucose (mg/dL)	94 ± 2
AHI (events/h)	1.7 ± 0.01
CRP (mg/dL)	0.26 ± 0.13

*Values expressed as mean ± standard error. BMI, body mass index; SBP, systolic blood pressure; DBP, diastolic blood pressure; LDL, low-density lipoprotein cholesterol; HDL-c, high-density lipoprotein cholesterol; AHI, apnea-hypopnea index; CRP, C-Reactive Protein*.

### Effects of acute intermittent HPX

The acute intermittent HPX effects on ventilatory, hemodynamic, vascular, and autonomic measures are shown in Table 2. Acute intermittent HPX reduced O_2_ saturation and increased HR, systolic BP, CO, PR, and HF band post HPX when compared to pre-HPX. However, there were no changes in end-tidal CO_2_ and diastolic BP from pre-HPX to post HPX 6. The LF band only increased in post HPX 5 and post HPX 6 when compared to pre-HPX (Table 2).

**Table 2:** **Acute intermittent hypoxia (HPX) on ventilator, hemodynamic and autonomic measurements**.

	**Pre-HPX**	**HPX 1**	**HPX 2**	**HPX 3**	**HPX 4**	**HPX 5**	**HPX 6**
O_2_ saturation (%)	99.1 ± 0.12	88.5 ± 1.05^*^	83.8 ± 1.44^*^	81.7 ± 1.46^*^	80.3 ± 0.47^*^	81.6 ± 1.36^*^	81.3 ± 1.72^*^
End-tidal CO_2_ (mmHg)	37.2 ± 0.02	36.8 ± 0.01	36.2 ± 0.05	36.6 ± 0.08	39.5 ± 0.07	37.6 ± 0.08	36.8 ± 0.1
Heart rate (beats/min)	70 ± 2.2	80 ± 3.3^*^	82 ± 3.3^*^	82 ± 3.0^*^	84 ± 3.0^*^	83 ± 3.1^*^	82 ± 3.3^*^
Systolic BP (mmHg)	125 ± 3.9	135.6 ± 6.3^*^	137 ± 6.6^*^	133.8 ± 5.7^*^	133.9 ± 5.7^*^	133.3 ± 5.6^*^	136.1 ± 5.5^*^
Diastolic BP (mmHg)	75 ± 2.2	75 ± 2.1	75 ± 1.7	74 ± 1.4	73 ± 1.3	74 ± 2	76 ± 2.2
Cardiac output (L/min)	5.59 ± 0.2	6.3 ± 0.24^*^	6.5 ± 0.28^*^	6.60 ± 0.29^*^	6.7 ± 0.29^*^	6.5 ± 0.33^*^	6.4 ± 0.33^*^
PR (dyn/sec/cm^5^)	1.00 ± 0.03	0.91 ± 0.04	0.90 ± 0.04^*^	0.88 ± 0.05^*^	0.86 ± 0.05^*^	0.88 ± 0.01^*^	0.85 ± 0.06^*^
LF (nu)	45.6 ± 3.3	59.6 ± 5.2	59.3 ± 5	60.2 ± 3.5	63.7 ± 5.4	65.4 ± 3.5^*^	68.9 ± 3.9^*^
HF (nu)	54.3 ± 3.3	40.3 ± 5.2^*^	40.6 ± 5^*^	39.7 ± 3.5^*^	36.2 ± 5.4^*^	34.5 ± 3.5^*^	29.7 ± 3.9^*^

Values expressed as mean ± standard error. HPX, intermittent hypoxia; SBP, systolic blood pressure; DBP, diastolic blood pressure; PR, peripheral resistance; LF, low frequency; HF, high frequency.

* *P <0.05 vs. Pre-HPX*.

#### Effects of acute intermittent HPX on PMNs and PBMCs

**Figure 2:**
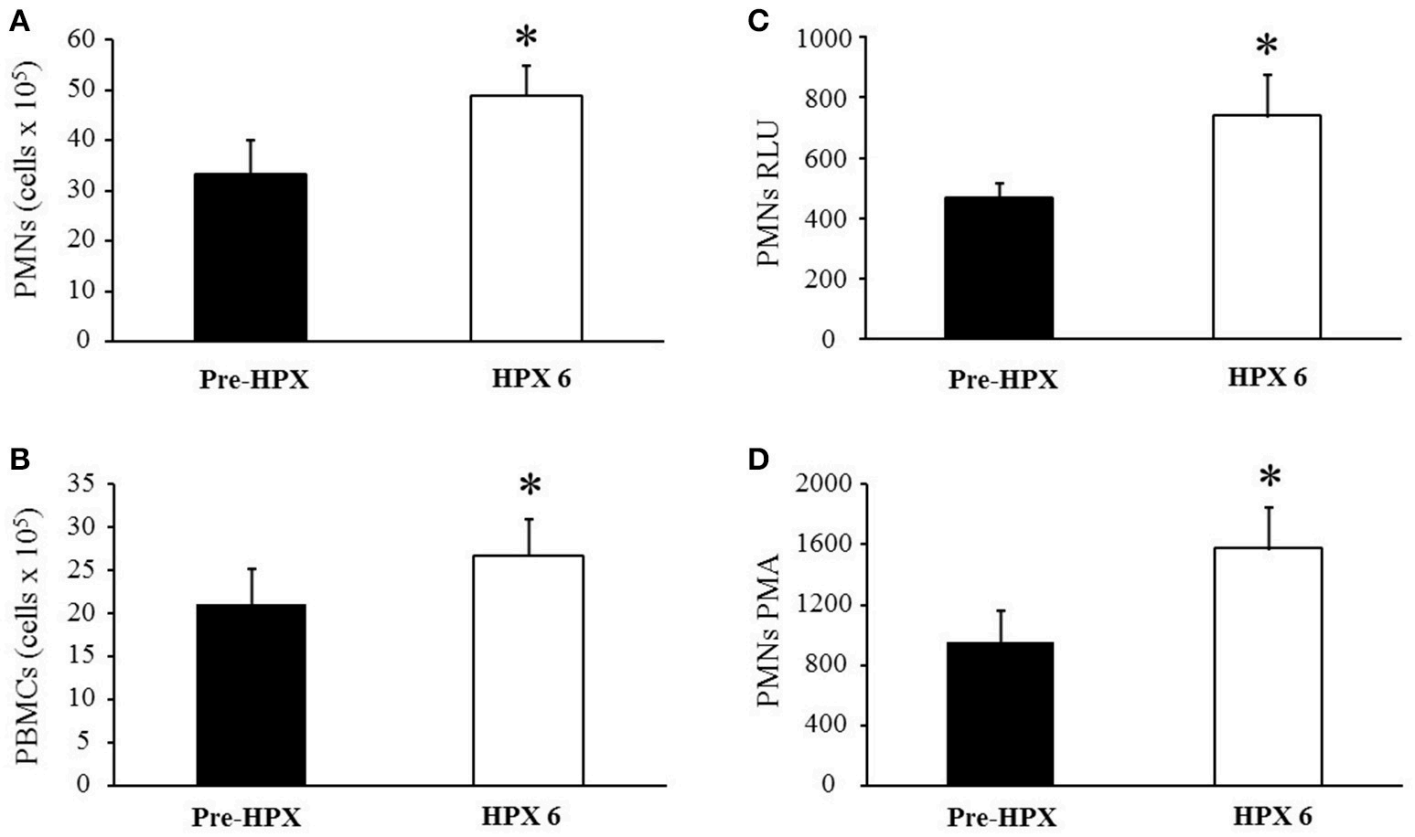
**(A)** Polymorphonuclear neutrophils (PMNs); **(B)** peripheral blood mononuclear cells (PBMCs); **(C)** polymorphonuclear in relative luminescence units (PMNs RLU); and **(D)** polymorphonuclear in relative phorbol myristate acetate (PMNs PMA). ^*^P < 0.05 vs. Pre-HPX.

Effects of acute intermittent HPX on PMNs and in PBMCs are shown in Figures 2A,B respectively. PMNs increased (34 ± 6.7–49 ± 6 cells × 10^5^; *P* = 0.008) from pre-HPX to post HPX 6 (Figure 2A). Similar results were found for PBMCs, which increased (21.5 ± 4.2–26.7 ± 4.4 cells × 10^5^; *P* = 0.03) from pre-HPX to post HPX6 (Figure 2B).

#### Effects of acute intermittent HPX on PMNs in relative luminescence units (RLU) and on PMNs in relative phorbol myristate acetate (PMA)

The effects of acute intermittent HPX on superoxide generation on PMNs-RLU and after the addition of 16 ng of phorbol 12-myristate 13-acetate (PMA) are shown in Figures 2C,D, respectively. Superoxide levels in PMNs RLU increased (470 ± 50–741 ± 135; *P* < 0.05) from pre-HPX to post HPX 6 (Figure 2C). The addition of PMA led to an increase in superoxide levels in PMNs harvested (1228 ± 247 vs. 1162 ± 265, *P* < 0.05) from pre-HPX to post HPX 6 (Figure 2D).

**Figure 3:**
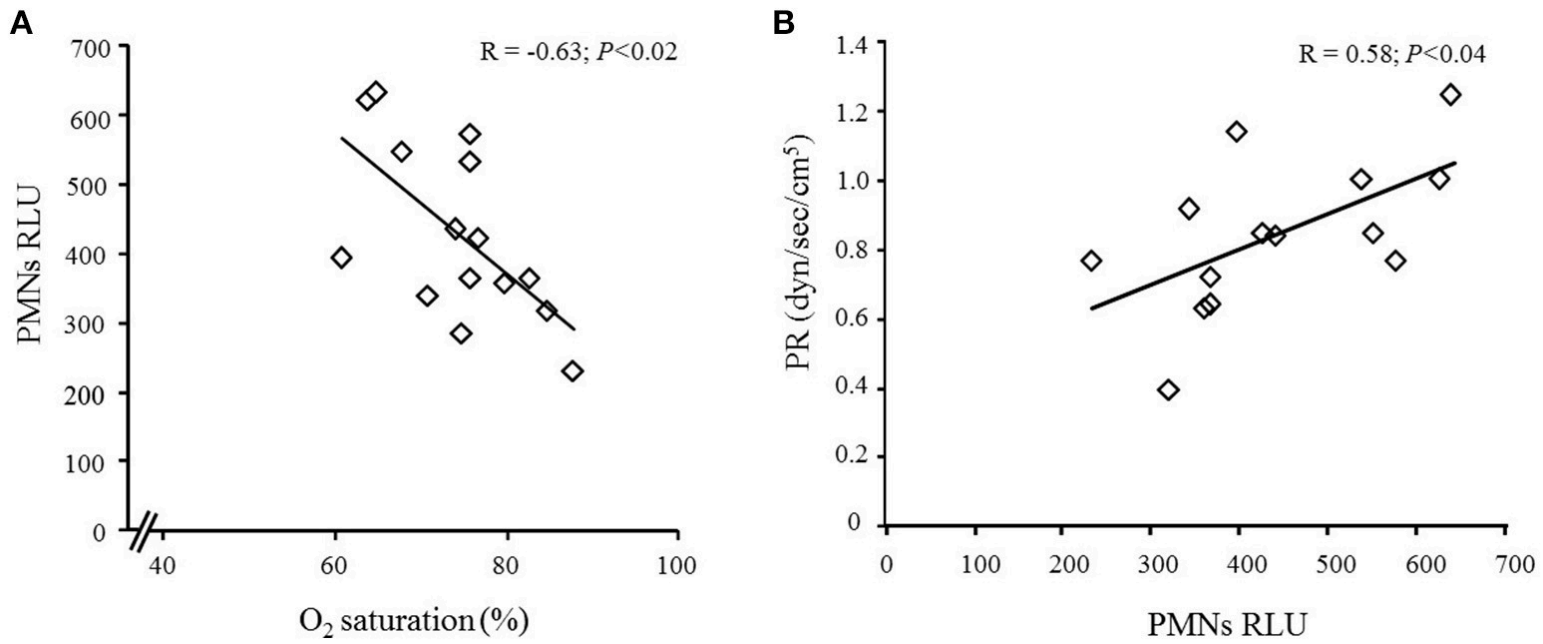
**(A)** Pearson correlation between O_2_ saturation and polymorphonuclear in relative luminescence units (PMNs RLU) in post HPX 6; and **(B)** Pearson correlation between PMNs RLU and peripheral resistance (PR) in post HPX 6.

Further analysis showed significant association between O_2_ saturation in HPX 6 and PMNs RLU post HPX 6 (*R* = −0.62, *P* = 0.02; Figure 3A). We also found an association between peripheral resistance (PR) in HPX 6 and PMNs–RLU post HPX 6 (*R* = −0.58, *P* = 0.04; Figure 3B).

#### Effects of acute HPX on heart rate variability

**Figure 4:**
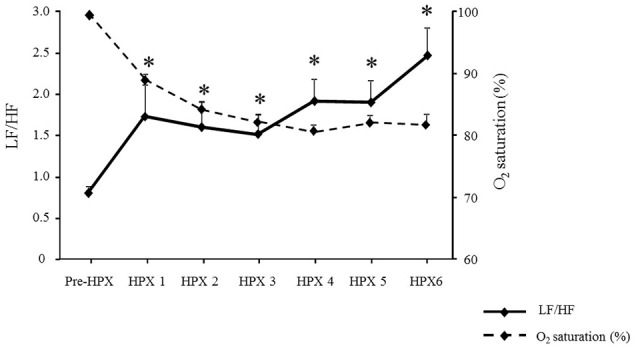
**Effects of the protocol of acute intermittent hypoxia (Pre-HPX and post HPX 1–6) in O_**2**_ saturation and on sympathovagal balance (LF/HF)**. ^*^P < 0.05 vs. Pre-HPX.

Acute intermittent HPX caused a significant increase in the sympathovagal ratio (LF/HF index) when compared to pre-HPX (Figure 4). The LF/HF index increased from 0.91 ± 0.11 in pre-HPX to 2.85 ± 0.4 in post HPX 6 (Figure 4). Interestingly, we found a strong association between O_2_ saturation in post HPX 6 and LF/HF in post HPX 6 (*R* = −0.79, *P* = 0.02).

## Discussion

The present investigation explores the role of intermittent HPX, a major component of OSA, in hemodynamic and autonomic responses and in the mobilization and activation of immune cells even in acute response in healthy subjects. The novel finding of our study is that acute exposure to intermittent HPX promoted not only hemodynamic and autonomic impairment in healthy volunteers but also increased superoxide generation in neutrophils.

The major implication of these findings is that intermittent HPX, in the absence of other pathophysiological changes, may be an important mediator in the genesis of oxidative stress in healthy individuals. In fact, in the present study, we found association between O_2_ saturation and polymorphonuclear in relative luminescence units (PMNs RLU) and the association between PMNs RLU and peripheral resistance (PR) in response to acute intermittent HPX. However, our results have to be interpreted with caution, because other potential mechanisms could be involved. In addition, a simple correlation between oxygen reactive species and a biological outcome does not always could be interpreted to causality.

The effects of intermittent HPX on several pathways have mainly been explored in animal models of OSA. Several studies have indicated that HPX is related to increased sympathetic activation and BP (Fletcher et al., 1992; Trombetta et al., 2013), as well as to inducing inflammation (Tam et al., 2007), oxidative stress (Jun et al., 2008), insulin resistance (Iiyori et al., 2007), dyslipidemia (Li et al., 2005; Drager et al., 2011a), and atherosclerosis (Savransky et al., 2007). The harmful effects of intermittent HPX have been demonstrated in autonomic and hemodynamic impairment (Foster et al., 2010; Gilmartin et al., 2010; Tamisier et al., 2011) and in insulin resistance (Iiyori et al., 2007). Our findings extend the previous evidence in humans, suggesting that intermittent HPX could trigger superoxide production even in the absence of diseases.

It is well-established that oxidative stress is implicated in vascular dysfunction and atherosclerosis. Several cardiovascular risk factors, such as hypertension, hypercholesterolemia, and diabetes mellitus, stimulate the production of superoxide in the vascular wall (Tousoulis et al., 2011). Accumulated evidence suggests that OSA is associated with increased oxidative stress and endothelial dysfunction. Lavie (2003) and Monneret et al. (2010). Indeed, OSA seems to directly affect the vascular endothelium by promoting inflammation and oxidative stress, while decreasing NO availability and repair capacity (Jelic et al., 2008), which may ultimately contribute to atherosclerosis (Drager et al., 2011b). Our results seems to corroborate these previous investigations. Although responses between health and the chronic “disease” state may be very different, the present study brings to the light the role of intermittent HPX, a hallmark of OSA, in healthy individuals. We found that acute intermittent HPX not only significantly increases the number of polymorphonuclear and peripheral blood mononuclear cells but also augments superoxide generation in polymorphonuclear cells. The precise mechanisms by which intermittent HPX induces oxidative stress through polymorphonuclear cells are yet to be determined. It has been postulated that the episodic hypoxia in OSA leads to the increased production of O_2_ and other reactive oxygen species molecules via several enzymatic pathways. For instance, upon stimulation, activation of NADPH oxidase complex in the endothelium and in neutrophils produces a burst of superoxide anions, contributing to oxidative stress and the onset of inflammatory processes (Lavie, 2003). We also cannot rule out the effect of the adrenergic activation triggered by intermittent HPX, as observed in our study on polymorphonuclear cells and oxidative stress. Indeed, several models of stress induction have demonstrated the onset of leukocytosis in response to catecholamines. Iversen et al. (1994) have found an increase in lymphocytes (220%) and neutrophils (160%) in response to epinephrine injection in rats. This effect was quite rapid (5 min or less) and brief, returning to normal 25 min after injection (Iversen et al., 1994). An early rise in leukocyte count is also a feature of ischemia reperfusion injury, as previously demonstrated in experimental studies (Hallenbeck et al., 1986; Grøgaard et al., 1989). Regarding oxidative stress, the role of epinephrine and adrenergic agonists in triggering oxidative stress in cell culture models (Costa Rosa et al., 1992) and animal models has been reported (Zhang et al., 2005). Therefore, the experimental model of acute hypoxia/reoxygenation presently described is capable of inducing a change in autonomic balance resulting in a predominance of sympathetic activity and an increase in white blood cell counts and oxidative stress, resembling several phenomena related to ischemia-reperfusion injury.

Despite these observations, we cannot exclude the possibility that oxidative stress induced by intermittent HPX may, indeed, be a potential trigger for sympathetic activation and increased BP. Evidence in animal models has suggested that increased oxidative stress in the brain, possibly via the activation of NADPH oxidase, may contribute to the progression of hypertension through central sympathoexcitation (Chan et al., 2009; Nagae et al., 2009). These important issues should be further explored in future experiments.

### Limitations

Our results have to be interpreted with caution. We cannot assume that in OSA patients we would find similar results because possible disparity regarding the chronic and acute effect could interfere in the responses. In addition, we have to address others important limitations.

Regarding superoxide generation in neutrophils determined by relative luminescence units, a large debate exists in the literature about the sensitivity of this assay and the use lucigenin. Indeed, although this assay presents a limitation due to the fact that lucigenin radical undergoes redox cycling with oxygen and generates superoxide, it has been widely used to measure kinetics productions of superoxide by neutrophils. We had previously reported that the assay conditions herein described are more appropriate than other techniques used to measure ROS production (Hatanaka et al., 2006). Luminol-amplified chemiluminescence, cytochrome c, hydroethidine, and phenol red assays also have limitations. The presence of antioxidant/oxidant molecules, enzymes such as neutrophil myeloperoxidase and even oxygen impairs the measurements of ROS production through the mentioned assays.

Other several limitations in our study that merit discussion. First, the duration of each cycle of intermittent HPX does not precisely mimic the duration of the sleep apnea events observed in OSA patients. In addition, the acute intermittent HPX protocol was brief and limited to only 30 min (6 cycles for 5 min). Additional exposure would involve substantial participant burden and could have untoward effects. Second, intermediate mechanisms were crudely assessed, using HR variability rather than microneurography for the assessment of sympathetic activity. Third, the acute intermittent HPX was induced during wakefulness and not during sleep. The decision to characterize hemodynamics, autonomic responses and oxidative stress during wakefulness was based on the awareness that hypoxic exposure during sleep would induce a range of pathophysiological abnormalities, including periodic breathing and sleep disruption. Thus, to characterize the isolated effects of intermittent HPX, the experiment was undertaken during wakefulness. Fourth, the experimental paradigm focused strictly on the effects of intermittent HPX and did not include other pathophysiological concomitants of OSA, such as hypercapnia or asphyxia. Finally, our study lack of a control group not exposed to intermittent HPX. The pre HPX measurements was used to identify the effects of acute intermittent HPX on superoxide generation.

In conclusion, acute exposure to intermittent HPX promoted autonomic impairments as well as increased oxidative stress. These data reinforce the role of intermittent HPX in superoxide generation on neutrophils, which may lead to an impairment in peripheral vascular resistance as observed in patients with obstructive sleep apnea.

## Author contributions

The specific contributions for each authors are: GA, EK, FC-C. Conception design of the work, the acquisition, analysis, interpretation of data for the work, drafting the work and revising it critically for important intellectual content. IT, FC, EH, RC, CM, MI, JB. Interpretation of data for the work; drafting the work, revising it critically for important intellectual content. All above authors read and approved the final version to be published; and all authors agree with all aspects of the work in order to ensure the accuracy and the integrity of the work.

## Disclosures

This was not an industry-supported study. The work was performed at the Heart Institute (InCor), University of São Paulo Medical School, São Paulo, Brazil and was supported by Fundação Zerbini. This study was supported by Fundação de Amparo a Pesquisa do Estado de São Paulo (FAPESP # 04/0222). FXC (FAPESP#2015/17533-6 and #2016/16831-7), RC (FAPESP 2010/02963-2, CNPq 303853/2015-3 and Guggenheim Foundation), CM (CNPq 442374/2014-3 and FAPEMA UNIVERSAL-00358/15 - Edital 40/2014).

### Conflict of interest statement

The authors declare that the research was conducted in the absence of any commercial or financial relationships that could be construed as a potential conflict of interest.
